# Introducing an integrated intermediate care unit improves ICU utilization: a prospective intervention study

**DOI:** 10.1186/1471-2253-14-76

**Published:** 2014-09-06

**Authors:** Barbara CJ Solberg, Carmen D Dirksen, Fred HM Nieman, Godefridus van Merode, Graham Ramsay, Paul Roekaerts, Martijn Poeze

**Affiliations:** 1 Staff department of Quality and Safety, Maastricht University Medical Center, P. Debyelaan 25, Maastricht, HX 6229, The Netherlands; 2 Clinical Epidemiology & Medical Technology Assessment (KEMTA), Maastricht University Medical Center, Maastricht, The Netherlands; 3 Department of Health Organisation, Policy and Economics (BEOZ), University of Maastricht, P.O. Box 616, Maastricht, MD 6200, The Netherlands; 4 Department of Surgery, Maastricht University Medical Center, Maastricht, The Netherlands; 5 Department of Intensive Care Medicine, Maastricht University Medical Center, Maastricht, The Netherlands; 6 Regent House, Mittre Way, Battle, East Sussex, UK

**Keywords:** Intensive care, Efficiency, Intermediate care, Resource allocation, Hospital bed capacity

## Abstract

**Background:**

Improvement of appropriate bed use and access to intensive care (ICU) beds is essential in optimizing utilization of ICU capacity. The introduction of an intermediate care unit (IMC) integrated in the ICU care may improve this utilization.

**Method:**

In a before-after prospective intervention study in a university hospital mixed ICU, the impact of introducing a six-bed mixed IMC unit supervised and staffed by ICU physicians was investigated. Changes in ICU utilization (length of stay, frequency of mechanical ventilation use), nursing workload assessed byTISS-28 score, as well as inappropriate bed use, accessibility of the ICU (number of referrals), and clinical outcome indicators (readmission and mortality rates) were measured.

**Results:**

During 17 months, data of 1027 ICU patients were collected. ICU utilization improved significantly with an increased appropriate use of ICU beds. However, the number of referrals, readmissions to the ICU and mortality rates did not decrease after the IMC was opened.

**Conclusion:**

The IMC contributed to a more appropriate use of ICU facilities and did result in a significant increase in mean nursing workload at the ICU.

## Background

The question of appropriateness of patient allocation for admission to and discharge from intensive care units (ICUs) is very pertinent [1]. Lack of ICU capacity may lead to patients being refused and may lead to inadequate care on the general ward. In addition, early discharge of ICU patients to general wards to reduce ICU patient load could lead to an increased rate of ICU readmissions and mortality [2,3]. This has raised public and political concern about the provision of critical care. The scarcity of critical care resources has resulted in suggestions to improve ICU utilization. A more effective triage reduces the number of inadequately allocated patients [4-6]. Previous studies showed that 35-40% of ICU patients could be treated at a lower level of care [7,8]. An intermediate care unit (IMC) can provide care for patients who do not require intensive care support, but need a higher level of nursing care that cannot be provided on the general ward. The IMC concept was suggested as a strategy to promote earlier discharge from ICU, facilitate patient re-allocation, decrease costs and prevent unnecessary ICU readmissions [9-11]. However, these studies were mainly retrospective. To investigate the impact on hospital care resources of introducing an IMC unit, we designed a prospective pre-test post-test intervention study.

The objective of this study was to investigate whether introducing an IMC unit as an integrated part of the daily ICU care would result in an improved ICU utilization, by comparing the periods before and after the opening of the IMC unit. Our hypothesis was that instituting an IMC unit leads to a decreased percentage intermediate care patients on the ICU, decreased inappropriate bed use on overflow wards, improved ICU utilization and improved outcome.

## Methods

### Design

The study was designed as a comparative longitudinal study that compared clinical and hospital data of patients who were admitted to the ICU before (pre-IMC period) and after (IMC period) introduction of the IMC. The total study period lasted 17 months, the pre-IMC period was 9 months (274 days) and the IMC period was 8 months (245 days) after a training period of one month. The Institutional Review Board of the Maastricht University Medical Center, Maastricht, The Netherlands approved the study. The requirement of informed consent was waived because the IMC was included in the usual care and no extra variables had to be collected.

### Patient population

The study population consisted of two groups of patients who were admitted to the ICU before and after the opening of the IMC. All patients admitted to the ICU were consecutively enrolled (*n* = 1027). A total of 548 patients during the pre-IMC period and 479 patients during the IMC period were included.

### Setting

The IMC was opened at University Hospital Maastricht as an integrated subunit directly adjacent to the medical-surgical ICU. The IMC had six beds in an open concept without isolation facilities. The general ICU was divided in two units, one of eight beds and one of nine beds. After the IMC had been opened, one ICU bed was closed (reducing the total to 16). Mechanical ventilation was available at all the ICU beds. The IMC was the entire study period only a step-down facility and patients were not directly referred to the IMC without prior ICU stay. Usually the patients with the lowest need for care were transferred to the IMC, while the new admission was admitted to the ICU for workup and stabilisation. After these goals were achieved this patient could then be transferred (the same or the next day) to the IMC. The ICU and the IMC were supervised and staffed by the same team of critical care physicians, who were available in the ICU and IMC 24 h/day, 7 days/week. To optimise the efficiency of the IMC, bed management was placed under the supervision of the medical ICU/IMC team [12]. The nursing staff for the IMC was a newly engaged team and was supplemented with one ICU nurse per shift. The nursing team were given a special training course. The ratio of nurses to patients in the IMC was 1:2 versus 1:1 in the general ICU and 1:5 in the wards on the day shift.

Intermediate care was defined as a level of care between intensive care and care on the general ward. Admission and discharge criteria for the ICU and IMC were based upon the criteria defined by Knaus et. al and Keenan et al. [13,14]. Identification of ICU patients was based upon interventions that could not be performed outside the critical care unit. These interventions were classified as Active Treatment according to Therapeutic Intervention Scoring System (TISS-28) variables such as mechanical ventilation and atrium monitoring. Non-active treatment variables represented interventions that could be carried out in IMC [15].

Cardiac patients were admitted to other separate specialised ICUs (cardiac ICUs) and were not included in the standard ICU capacity. The cardiac ICU and the 24 h-post-anesthesia care unit (PACU) served as overflows for general ICU patients when the general ICU was fully occupied. Neurosurgical and cardiac-surgical patients were transferred from the ICU to a specialised intermediate care integrated into the general ward.

### Data collection

The data collected for each ICU patient included the following: age, gender, length of stay (ICU, IMC), mortality (ICU and general ward), type of stay (nonsurgical versus surgical), nursing workload, number of days of mechanical ventilation, severity of illness and diagnostic category. Parameters were divided into four categories: 1) Utilization of ICU, 2) inappropriate bed use, 3) accessibility of the ICU, and 4) clinical outcomes. Variables relating to utilization of ICU comprised the following: length of stay of ICU and IMC, number of days of mechanical ventilation, nursing workload during ICU and at ICU discharge. Inappropriate bed use for the ICU and IMC was measured as active versus non-active treatment criteria and the level of nursing care. Inappropriate admission to the ward was defined on predetermined criteria based upon an early warning system [16]. Accessibility of the ICU was measured by ICU referrals. Variables relating to clinical outcome were ICU readmissions and mortality. Explanation of these variables follows.

### Patient demographics and ICU admission characteristics

Variables relating to demographics were collected for every ICU and IMC patient. Severity of illness was measured by the Acute Physiology and Chronic Health Evaluation (APACHE) II scoring-system [17]. Diagnostic category was defined according to the diagnosis classification in APACHE II; neurological, neuro-surgical, respiratory, gastro-intestinal, cardiovascular, multi-trauma, sepsis, renal, metabolic and haematological. The ICU admission characteristic was measured by the nursing workload on admission day of the ICU. The simplified Therapeutic Intervention Scoring System (TISS-28) was used as a validated assessment of the nursing workload, weighting and summarizing critical care nursing interventions (one to eight points per intervention) based on the complexity of expertise required [18,19]. TISS-28 scores were routinely calculated on a daily basis as cumulative 24-hr scores for the ICU and the IMC unit. In the demographic data analysis, each patient counted as a single admission, to prevent readmissions from inappropriately affecting the results on age, gender and mortality. All other data were analysed on an individual admission basis.

### Efficiency parameters

In this study, changes in utilization between the pre-IMC and IMC periods were investigated by recording capacity of ICU, inappropriate bed use, accessibility of the ICU and clinical outcome. Utilization of the ICU was measured as ICU length of stay, number of days of mechanical ventilation, TISS-28 score during ICU stay and TISS-28 score on the ICU discharge day and whether patients had or did not have a TISS-28 score < 20 at discharge from the ICU.

The number of inappropriate stays in the ICU, cardiac-surgical ICU, PACU and general wards measured inappropriate bed use. At the ICU, it was the medical and nursing staff who determined whether a patient’s ICU stay was appropriate or inappropriate, because of delayed discharge or admission without real ICU indication, based on the Active and Non-active treatment criteria according to TISS-28 [13-15]. Another way to determine an ICU patient was appropriate or inappropriate was by categorising the TISS-28 score into the level of care that patients require. Several studies have shown that TISS-28 scores could differentiate between ICU and IMC [20]. The total cumulative score classifies patients into Class 1, i.e. those who do not need intensive care observation (low care; <10 points); Class 2, i.e. those who need intermediate care (intermediate care; 10–19 points); Class 3, i.e. those who need high care (high care; 20–39 points) or Class 4, i.e. those who need intensive care (intensive care; ≥40 points) [20,21]. Inappropriate ICU used was measured as the number of patients on Non-active treatment and TISS-28 < 20 points. Inappropriate use of the cardiac-surgical ICU and PACU was measured by recording daily numbers of general ICU patients when the overflow function was used. Each day, the nursing staffs of four general wards indicated the number of intermediate care patients present on the general wards, based upon Early Warning System.

Accessibility in this study was defined as the number of patients refused for admission to the ICU assessed as with and without a real ICU indication. Referrals were assessed daily by the medical staff responsible for the admission and discharge policy. Medical staff determined if a referral had a correct indication for ICU admission based on admission or discharge criteria derived from the guidelines of the Task force of the American College of Critical Care Medicine [13]. Prior to the installation of the IMC, patients in need of intermediate care level being refused due to shortage of beds on the ICU were usually left on the ward or transferred to an overflow unit, such as the PACU unit, when having a real ICU indication. This was also performed after the opening of the IMC by the medical staff in charge of the ICU.

Finally, clinical outcome indicators were measured by readmission to the ICU and hospital mortality after ICU and IMC discharge. Readmissions were defined as taking place within 48 hours during the same hospital stay.

### Statistics

The demographics analysis used the patient as the unit of analysis, while the analysis of utilization parameters was based on characteristics of the ICU stay or hospital admission. Continuous outcome variables were first tested for normality of statistical distribution by the Kolmogorov-Smirnov test. If they were distributed normally, means and standard deviations were calculated and the Student independent groups *t*-test was performed to test differences between the pre-IMC and IMC periods. If not, the Mann–Whitney test was used for this purpose. Categorical data were calculated as frequencies and percentages, and the log-likelihood chi-squares test was used to test outcome differences between the two periods. A p-value of less than 0.05 was regarded as statistically significant. All data analyses were performed with SPSS version 16.0 (Statistics Package for the Social Sciences, SPSS Inc. Chicago, Illinois 60606).

## Results

### Patient population

**Figure 1:**
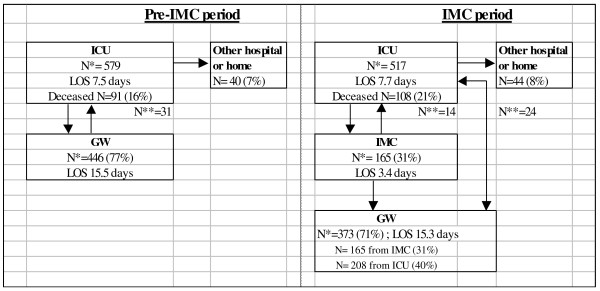
**Patient flow.** N*, number of admissions; N**, number of readmissions; ICU, Intensive care unit; IMC, intermediate care unit; GW, general ward; LOS, length of stay.

Figure 1 shows the patient flow expressed as the number of admissions to the ICU, IMC unit and general wards (GW), ICU readmissions, ICU discharges, mean length of ICU stay (LOS) and the number of deceased ICU patients during the pre-IMC period and the IMC period. During the pre-IMC period, the LOS of ICU patients was 5.8 days, and 446 were referred to the general ward (77%). During the IMC period, the LOS of ICU patients was 7.0 days, and 165 patients (31%) were referred to the IMC unit and 208 patient (40%) of the ICU patients was referred directly to the general ward. The LOS of the patients admitted to the IMC unit was 3.4 days. Table 1 summarizes demographic and admission characteristic for ICU patients during the two periods. Characteristics of 548 consecutive patients in the pre-IMC period were compared with those of 479 consecutive patients in the IMC period. There were no significant differences in demographic data between ICU patients in the pre-IMC and IMC periods. The average TISS-28 score on ICU admission increased significantly (p = 0.005) after the IMC unit was opened.

**Table 1:** Patient demographics and ICU admission characteristics

	**Pre-IMC period**	**IMC period**	**p-value**
Number of patients	548	479	
Number of stays	579	517	
ICU days	4083	3692	
Number of beds	17	16	
Gender, (% male)	58%	60%	0.57*
Age, (mean, ±SD)	55 (18.1)	56 (18.2)	0.80**
% surgical	60.2%	61.5%	0.59*
*Diagnostic category*			0.50*
Neuro-neurosurgical	26.5%	29.5%	
Respiratory	22.4%	19.8%	
Gastro-intestinal	19.4%	19.2%	
Cardiovascular	12.7%	14.7%	
Multi-trauma	11.2%	8.4%	
Sepsis	4%	4.2%	
Metabolic/renal	3.6%	3.4%	
Haematological	0.2%	0.8%	
APACHE II (mean, ±SD)	19.3 (8.1)	19.6 (7.6)	0.43**
TISS-28 on ICU admission day (mean, ±SD)	28.7 (10.6)	30.6 (11.0)	0.005**

*chi-square tests; **Mann Witney tests.

### Efficiency parameters

Changes in utilization after the introduction of the IMC unit are shown in Table 2. Inappropriate use of the ICU, PACU and general ward decreased significantly. The total number of patients admitted inappropriately on the overflow units decreased from 30.8% to 18.6% (P < 0.0001). Both the number of active versus non-active treatment patients and the number of active versus non-active treatment days decreased significantly after opening the IMC unit (p < 0.01). In addition, the number of days with patients having a TISS score < 20 decreased significantly (p < 0.01). ICU acuity increased after the opening of the IMC, as indicated by a significant increase in the mean TISS-28 during ICU stay and a significant increase in the number of days of mechanical ventilation at the ICU. In the IMC period, ICU patients who were discharged were in need of a higher level of care than in the pre-IMC period, as indicated by a significantly decreased percentage of patients with a TISS-28 below 20 points and an increased mean TISS-28 score at ICU discharge. Importantly, the number of patients defined as inappropriate admissions to the PACU, as overflow unit, decreased significantly by 50% (p = 0.02). In addition, the number of patients and the number of days of IMC patients inappropriately admitted to the general ward decreased significantly (p < 0.01). However, the number of readmissions to the ICU did not change during the IMC period. The number of patients refused during the IMC period was similar to that of the pre-IMC period. In addition, the number of refused patients who were indicated for re-admission was similar in both periods (5/31 versus 2/38 for pre-IMC and IMC period respectively, p = 0.26). No change was found in the mean length of ICU stay (LOS). A slight decrease in referrals was found (2.5%), but this was not statistically significant. The mortality rate of patients at the ICU increased significantly during the IMC period (p = 0.02). The IMC was deemed not applicable for terminal patients or for palliative care. So patients who were determined to be dying stayed in the ICU or transferred to the general ward.

**Table 2:** ICU utilization parameters

**Parameters**	**Pre-IMC period**	**IMC period**	**P-value**
**(No. and % of total ICU population)**	**N = 548**	**N = 479**	
**1. Utilization of ICU**			
ICU length of stay (mean, ±SD)	7.5 (14.5)	7.7 (12.4)	0.74**
IMC length of stay (mean, ±SD)	-	3.4 (4.5)	
Mechanical ventilation (% days)	71.9%	90.6%	< 0.001**
TISS-28 during ICU stay (mean, ±SD)	26.4 (8.7)	29 (9.5)	< 0.001**
TISS-28 on discharge day (mean, ±SD)	23.9 (9.2)	26.0 (10.1)	< 0.001**
TISS-28 on discharge day < 20	37.1%	28.1%	0.002**
**2. Inappropriate bed use**			
Inappropriate ICU use			
a. Active-Non active treatment			
Patients	69 (11.9%)	7 (1.3%)	< 0.01*
Days	265 (3.2%)	19 (0.01%)	
b. TISS-28 < 20			
Days	764 (18.7%)	357 (9.7%)	<0.01*
**Inappropriate cardiac ICU use**			
Patients	27 (4.9%)	17 (1.3%)	0.25*
Days	107 (2.5%)	50 (1.4%)	
**Inappropriate PACU use**			
Patients	19 (3.2%)	8 (1.5%)	0.02*
IMC patients in general ward			
Patients	123 (21.1%)	64 (12.2%)	< 0.01*
Days	267 (6.1%)	153 (4.3%)	
**3. Accessibility of the ICU refused ICU admissions**			
Total number of patients	110 (20%)	77 (16%)	0.13*
Of whom real ICU indication	58 (10.6%)	37 (7.7%)	0.12*
**4. Clinical outcome readmissions (total)**			
	31 (5.2%)	38 (7.9%)	0.15*
Readmissions from general ward	31 (5.2%)	24 (4.6%)	0.56*
Of which readmission <48 h.	7 (1.2%)	3 (0.6%)	0.27*
Readmission from IMC	-	14 (2.7%)	-
**Mortality at ICU**	91 (16,6%)	108 (22.5%)	0.02*
**Mortality on general ward**	24 (4.4%)	20 (4.2%)	0.80*

*chi-square tests; **Mann–Whitney tests.

## Discussion

Providing intensive care to adequately selected patients is one of the greatest challenges in ICU care, since the limited resources on the intensive care unit will be quickly depleted by an inability to transfer intermediate care patients from the ICU to the general wards. This study demonstrated that the introduction of an IMC unit was associated with more appropriate utilization of ICU resources with a reduced inappropriate bed use of previously used overflow units.

Inappropriate bed use at the ICU was found to decrease after the opening of an IMC unit at our hospital, indicating that increased flow of patients out of the ICU to the IMC can provide a relative increase in bed capacity on the ICU. Previous studies have demonstrated that 35-40% of ICU patients could be treated or monitored at IMC units [6,7]. Comparing with historic study data, Fox et al. showed that the ICU bed occupancy rate for low-risk patients using an IMC unit as step-down unit decreased from 21.6 to 11.2% [22]. In our study, a significantly increased number of patients were correctly allocated to the ICU. Interestingly, inappropriate bed use at the previously used overflow units, such as the PACU, also decreased. Previously Kastrup et al. showed that the introduction of a PACU and the staffing with intensivists increased the number of patients on the ICU in need for prolonged (>7 days) stay [23]. More importantly, inappropriate bed use by IMC patients on general wards was also decreased by 50%. This reduced inappropriate bed use should improve the provision of adequate levels of care.

Our results confirm the hypothesis that an IMC was associated with improved utilization of intensive care support for the patients admitted to the ICU. In a study by Eachempati et al. the introduction of a step-down intermediate care unit resulted in a significant increase in the overall severity of the SICU population, although this study did not include the level of inappropriate bed use by e.g. the TISS-28 score [24]. Our study was the first to relate these changes. Zimmerman et al. mathematically excluded patients with a low risk of receiving intensive care treatment during their ICU stay from the population in need of intensive care treatment, and found TISS scores in the latter group that were similar to our data [8]. This finding is also supported by a study by Byrick et al., who found that TISS-28 scores of ICU patients decreased from 37.1 to 27.9 when an IMC unit was closed [10]. However, the TISS-28 and increased mechanical ventilation data may also be suggestive for a higher acuity patient population during the study period.

The introduction of the IMC unit did not change the length of stay at the ICU. Other studies have suggested that introducing an IMC would promote earlier ICU discharge and thereby reduce the length of stay at the ICU [2,3,10]. This notion, however, was not confirmed in our study. Several factors may explain the lack of expected LOS reduction. One of the reasons for this may the reduced a priori ICU capacity during the IMC period. In addition, IMC patients post discharge from the ICU in need of general ward bed could not always be transferred to a normal ward due to limitations in bed capacity on these wards. One other reason may be that inappropriate ICU patients with indications for IMC admission generally have a short LOS at the ICU. We found increases in TISS-28 scores during ICU stay, increased numbers of days of mechanical ventilation and increased ICU mortality in the IMC period, which indicates that splitting up the population into ICU and IMC patients led to the allocation of a more severely ill ICU population to the intensive care, so during the IMC period there was a higher acuity and census.

A number of comments need to be made on the interpretation of our observations. A significant increase in TISS-28 scores on the day of admission to the ICU may be considered to indicate that a sicker population was admitted to the ICU. On the other hand the TISS-28 may have limitations in detecting increases in level of workload on an individual basis, but in general is good in detecting differences in workload on a population base [20]. The reduction of the number of ICU patients primarily admitted to the overflow units, by step-down transfer of intermediate care patients to the IMC may also have increased the TISS-28 score on day of admission. Whether or not true remains debatable since all patients were still primarily admitted to the ICU without changes in refusal rate.

A confounding factor may be that the demand/supply ratio for ICU beds *per se* increased over time, which influences ICU utilization data, regardless of the effects of introducing an IMC unit. Indeed, the number of referrals from outside our hospital increased. In addition, after the IMC unit was opened, the number of ICU beds was reduced from 17 to 16. However, there was no increase in the number of patients refused. Addition analyses taking the reduction of one ICU bed into account did not change the outcome in terms of refusals. Interestingly, the percentage of readmissions was comparable to previously published data [25-27] and did not change in the study period (prior 5.5% versus 4.6% after opening the intermediate care unit).

Accessibility of the ICU was defined in this study by the intensivist on call, and was not based on predefined TISS or APACHE scores. This could have introduced selection bias of patients after the opening of the IMC unit and would thus in theory decrease the number of refused ICU admissions. However, the percentage of patients who were refused ICU admission was similar for the pre-IMC and IMC periods. A parallel trial using hospital-based randomization for the introduction of an IMC unit would reduce this bias, but would not be feasible at a single institution. In addition, it was chosen for this project not to use a step-up approach from the general ward to the intermediate care unit, due to limitations in staff numbers. Thus, although no step-up function to the IMC from the general ward was available, patients could be transferred from the general ward to the overflow units, such as the PACU and other medium care units, when ICU capacity was insufficient. Therefore we primarily admitted the patients primarily to the ICU and then as a step-down to the IMC unit. Most of the patients needed e.g. invasive monitoring, which was started at the ICU. After initial stabilisation, patients could be discharged to the IMC. In our organisation this was at that time a more logical approach. However, the use of a simultaneous approach for step-up of IMC patients could have increased the ICU utility further.

The efficiency of an IMC unit is intimately related to the staffing levels. Nursing workload at the IMC unit increased from the beginning till the end of the IMC period. Since there was no increase in physician man power for the increased patient load, this calls for concern as a potential negative impact of opening an IMC. As time went on, patients were admitted to the IMC unit with increasingly severe illness, and the occupancy rate of the IMC beds increased. A longer run-in period compared to the training period of one month might have improved the adequacy of allocation prior to the actual study period.

## Conclusion

In conclusion, the introduction of an IMC unit was associated with improved ICU utilization and more appropriate ICU and specialized unit use. In this study, there was no change in demand for ICU care or ICU LOS. This may have been a result of a higher acuity patient population after the opening of the IMC.

## Competing interests

The authors have declared that no competing interests exist.

## Authors’ contributions

Conceived and designed the study BCJS/CDD/GR, performed the study BCJS/CDD, analysed the data BCJS/CDD/FHMN/MP, wrote the paper BCJS/MP, all authors read and approved the final manuscript.

## Pre-publication history

The pre-publication history for this paper can be accessed here:

http://www.biomedcentral.com/1471-2253/14/76/prepub
